# Microbiota-Derived SCFAs Mediate the Synergistic Antidepressant Effects of Dajianzhong Decoction and Ketamine via FFAR2-NLRP3-IL-1β Signaling

**DOI:** 10.3390/ph19060877

**Published:** 2026-05-31

**Authors:** Wenting Zhang, Xin Guo, Jiping Zhang, Yuan-Wei Zhang, Chan Li

**Affiliations:** 1 School of Life Sciences, Guangzhou University, Guangzhou 510006, China; 2112314070@e.gzhu.edu.cn (W.Z.); 2112414109@e.gzhu.edu.cn (X.G.); 2 School of Traditional Chinese Medicine, Southern Medical University, Guangzhou 510515, China; zhangjp611@163.com

**Keywords:** depression, ketamine, DJZT, synergistic mechanism, SCFAs, FFAR2–NLRP3–IL-1β

## Abstract

**Background:** Ketamine is a rapid-acting antidepressant for major depressive disorder; however, its effects are short-lasting and associated with neurotoxic side effects. Thus, identifying strategies to prolong its antidepressant effects is of critical importance. It has been shown that Dajianzhong Decoction (DJZT) prolongs the antidepressant effects of ketamine through modulation of the gut microbiota, but the underlying mechanisms remain unclear. **Method:** Fecal microbiota transplantation, metabolomic profiling, pharmacological interventions, and behavioral approaches were employed together with a chronic unpredictable mild stress (CUMS) mouse model to investigate how microbiota-derived signals mediate the combined effects of DJZT and ketamine. **Results:** Microbiota from CUMS mice induced depressive-like behaviors in recipient mice, accompanied by reduced levels of short-chain fatty acids (SCFAs), decreased FFAR2 expression in the medial prefrontal cortex, and increased neuroinflammation and synaptic deficits. These alterations were reversed by microbiota from DJZT-plus-ketamine-treated donors. Notably, acetic acid and isobutyric acid were identified as key SCFAs restored by the combined treatment and were significantly associated with behavioral outcomes. Moreover, SCFA supplementation recapitulated these effects by activating FFAR2 and suppressing NLRP3–IL-1β signaling. Importantly, pharmacological inhibition of FFAR2 using GLPG0974 abolished the antidepressant-like, anti-inflammatory, and synaptic protective effects of the microbiota from DJZT-plus-ketamine-treated donors. **Conclusions:** These findings demonstrate that microbiota-derived SCFAs mediate the synergistic antidepressant effects of DJZT and ketamine via a central FFAR2-dependent mechanism involving suppression of neuroinflammation. This work highlights a potential role of the SCFA–FFAR2–NLRP3– IL-1β axis in influencing ketamine efficacy and points to microbiota-modulating strategies as a possible avenue for improving antidepressant therapy.

## 1. Introduction

Depression is a common mental disorder characterized by persistent low mood, loss of interest, and sleep disturbances, and it is associated with high rates of disability, suicide, and recurrence [1]. It imposes a major global health burden, affecting approximately 332 million people worldwide in 2021 [2]. In China, the lifetime prevalence of depressive disorders has been reported at 6.8%, with an estimated 95 million individuals impacted by depression-related conditions [3]. Therefore, the prevention and treatment of depression continue to be urgent public health imperatives.

Pharmacological intervention remains the primary treatment strategy for depression; however, conventional antidepressants—selective serotonin reuptake inhibitors (SSRIs)—are limited by treatment resistance, delayed onset of action, and frequent relapse, with approximately 30–40% of patients failing to respond [4,5]. In recent years, ketamine, as a novel rapid-acting antidepressant, has been demonstrated to be effective for SSRI treatment-resistant symptoms and for protection against relapse, thus distinguishing it from SSRIs and ushering in a new era of antidepressant drug development [6]. However, the antidepressant efficacy of ketamine is relatively short in duration, usually lasting no longer than one week, and its clinical application is severely limited by side effects such as psychotomimetic and dissociative symptoms and abuse potential. Thus, it is essential to explore adjunctive therapies for enhancing the antidepressant effects of ketamine and minimizing dosing frequency and potential adverse effects.

As the maintenance of ketamine’s antidepressant effects depends on restoration of lost spines [7], relapse may reflect impermanence of regrown synapses [8]. In this context, strategies aimed at prolonging the persistence of synapses could extend the efficacy of ketamine. Neuroinflammation, a key driver of synaptic loss and neuronal dysfunction, is critically involved in the onset and progression of depression [9,10]. Recently, both clinical and preclinical studies have emphasized that neuroinflammation in depression is closely associated with gut microbiota dysbiosis. Stress exposure induces alterations in gut microbiota composition and microbial metabolism, which can influence central immune and neural processes via the gut–brain axis [11,12]. Among these microbiota-derived metabolites, short-chain fatty acids (SCFAs), the major end products of bacterial fermentation in the gut, play important roles in maintaining intestinal barrier integrity, regulating neurotransmitter systems, modulating inflammatory responses, and influencing synaptic plasticity [13,14]. Therefore, elucidating how gut-derived metabolites, especially SCFAs, regulate neuroinflammation and synaptic plasticity may provide new insights into a mechanism for developing strategies to prolong the antidepressant efficacy of ketamine.

Traditional Chinese medicine (TCM) has unique advantages in the treatment of depression, including definite clinical efficacy, multi-target actions, holistic regulation, and relatively low toxicity. Moreover, TCM can synergize with conventional antidepressants to enhance therapeutic efficacy while reducing adverse effects. Accordingly, exploring TCM-based strategies to prolong the antidepressant effects of ketamine and mitigate its side effects is of great importance [15,16]. Dajianzhong Decoction (Dajianzhong Tang in Chinese, DJZT) is a classical herbal formula traditionally used for the treatment of gastrointestinal disorders. Clinically, it has been widely prescribed for digestive system diseases such as postoperative paralytic ileus, Crohn’s disease, and inflammatory bowel disease. Accumulating pharmacological evidence indicates that DJZT exerts anti-inflammatory and analgesic effects, modulates gastrointestinal function, enhances SCFA production, and regulates immune responses within the gastrointestinal tract [17,18,19,20]. Furthermore, our previous study demonstrated that a combined use of DJZT with ketamine markedly reshaped gut microbiota dysbiosis, suppressed serum inflammatory cytokines, and prolonged the antidepressant efficacy of ketamine in a chronic unpredictable mild stress (CUMS) mouse model. However, the molecular mechanism underlying this synergistic antidepressant effect remains largely unclear [21]. In the present study, we investigated whether an induced microbiota-derived anti-inflammatory action is necessary and sufficient to drive synergistic antidepressant effects in mice.

## 2. Results

### 2.1. Chemical Characterization of DJZT by UHPLC–OE–MS

DJZT consists of *Zingiber officinale* Rose, *Zanthoxyli bungeanum* Maxim., *Panax ginseng* C. A. Mey, and maltose. To characterize its chemical composition, we performed ultra-high performance liquid chromatography coupled with an Orbitrap Exploris 120 mass spectrometer (UHPLC–OE–MS) analysis. A total of 17 major compounds were identified in the DJZT extract in positive ion mode (Figure S1), and a total of 11 major compounds were detected in negative ion mode (Figure S2).

### 2.2. DJZT Prolongs the Rapid Antidepressant Action of Ketamine on Animal Behaviors

**Figure 1:**
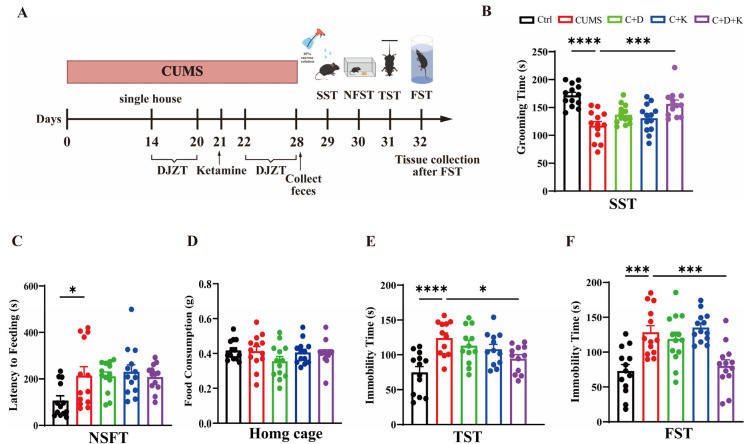
DJZT sustains ketamine-induced antidepressant responses in behavioral tests 7 days after a single ketamine injection. (**A**) Schematic diagram of the experimental design. Mice received DJZT (300 mg/kg, daily) from day 14 to day 28 and a single injection of ketamine (10 mg/kg) on day 21. CUMS mice received saline instead of drugs. Fecal samples were collected on day 28 for FMT. Behavioral tests were conducted day 29, including SST, NSFT, TST, and FST. (**B**) Grooming time during a 5 min observation period was recorded in SST. (**C**) Latency to feed was measured in NSFT performance. (**D**) The home cage food consumption within 20 min after NSFT. (**E**,**F**) Immobility time during the 2–6 min interval was quantified in TST and FST. Each bar represents the mean ± standard error of the mean (SEM.) (*n* = 13 mice per group). Experimental groups were defined as follows: Control (Ctrl), CUMS (CUMS), CUMS + DJZT (C+D), CUMS + ketamine (C+K), and CUMS + DJZT + ketamine (C+D+K). Statistical analysis was performed using one-way ANOVA followed by Tukey’s multiple comparisons test. * *p* < 0.05, *** *p* < 0.001, **** *p* < 0.0001. See Supplementary Tables S1-1 and S1-2 for detailed statistics.

In our previous study, we demonstrated that co-administration of ketamine with DJZT effectively sustained the antidepressant effects of ketamine in behavioral assessments conducted 7 days after a single ketamine injection [21]. Based on these findings, the present study employed a 7-day post-ketamine evaluation to further investigate this synergistic interaction (Figure 1A). Consistent with our prior observations, CUMS-induced behavioral abnormalities—including reduced grooming time in the sucrose splash test (SST) and increased immobility time in both the tail suspension test (TST) and forced swim test (FST)—were significantly ameliorated by the combined administration of ketamine and DJZT (Figure 1B,E,F). However, it had little effect on the latency to feed and home cage food consumption in the novelty-suppressed feeding test (NSFT) (Figure 1C,D). In contrast, ketamine or DJZT alone did not produce a behavioral improvement at this time point. Collectively, these findings indicate that DJZT prolongs the rapid antidepressant effects of ketamine.

### 2.3. Fecal Microbiota from Mice Treated with DJZT Plus Ketamine Alleviate Depressive-like Behaviors in Recipient Mice

**Figure 2:**
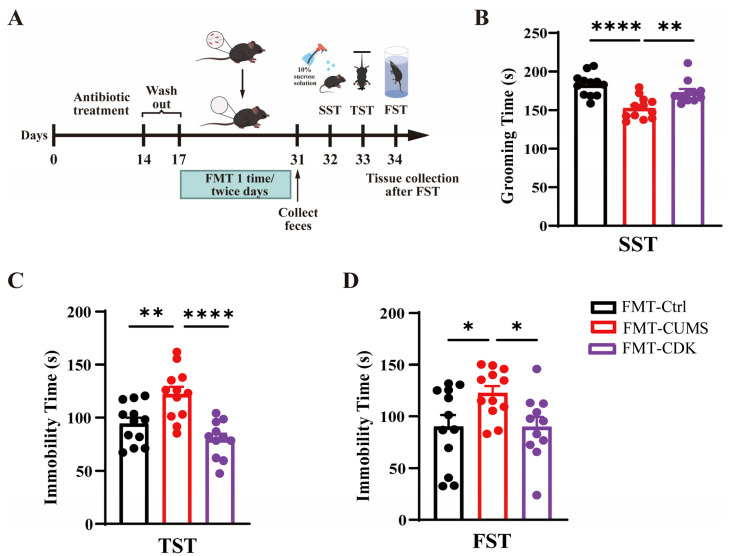
FMT from DJZT-plus-ketamine-treated donor mice mediates antidepressant effects in recipient mice. (**A**) Experimental design for FMT in antibiotic-cocktail-treated, pseudo-germ-free mice. Fecal samples were collected on day 31 and subjected to targeted metabolomic analysis. SST was conducted on day 32, followed by TST and FST. (**B**–**D**) The grooming and immobility time was measured in SST, TST, and FST. Each bar represents the mean ± SEM (*n* = 12 mice per group). Statistical analysis was performed using one-way ANOVA followed by Tukey’s multiple comparisons test. * *p* < 0.05, ** *p* < 0.01, **** *p* < 0.0001. See Supplementary Tables S2-1 and S2-2 for detailed statistics.

Previously, we demonstrated that co-administration of DJZT and ketamine markedly altered the gut microbiota composition in CUMS mice. In light of this observation, we hypothesized that gut microbiota remodeling may contribute to the synergistic antidepressant effects of the combined treatment. To test this possibility, an antibiotic cocktail was used to establish a microbiota-depleted state, administered ad libitum via drinking water. Fecal microbiota transplantation (FMT) was then performed to evaluate whether microbial alterations mediate behavioral performance (Figure 2A).

Microbiota from control, CUMS, and DJZT-plus-ketamine-treated donor mice were transplanted into the recipient mice that had been treated with antibiotic cocktail to establish a pseudo-germ-free model. Two weeks after FMT, recipient mice receiving microbiota from CUMS donors (FMT-CUMS) displayed significant depressive-like behaviors, including reduced grooming time in the SST and increased immobility time in both the TST and FST, compared with FMT-control (FMT-Ctrl) mice. Conversely, transplantation of microbiota from DJZT and ketamine co-treated donors (FMT-CDK) effectively reversed these deficits, restoring behavioral performance to levels comparable to controls (Figure 2B–D). Together, these results suggest that gut microbiota remodeling is a key mediator of the synergistic antidepressant effects of DJZT combined with ketamine.

### 2.4. DJZT in Combination with Ketamine Ameliorates CUMS-Induced Reductions in Fecal SCFAs

**Figure 3:**
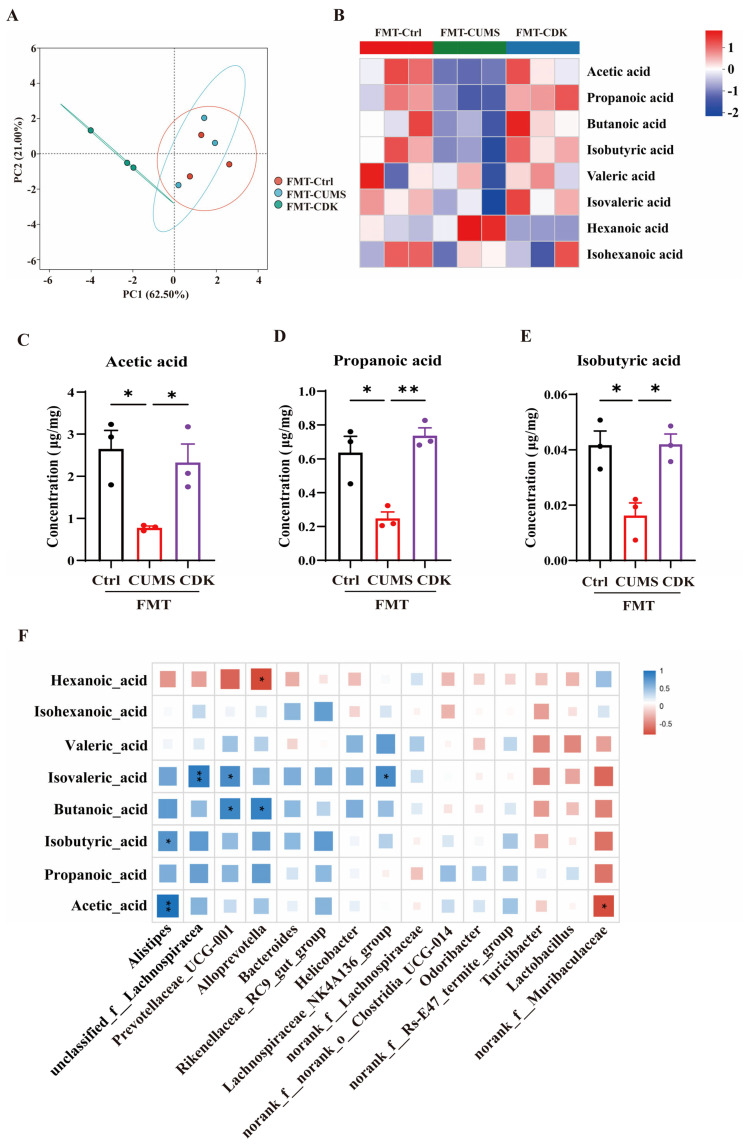
Fecal microbiota from DJZT-ketamine-treated mice increases SCFA levels in feces. (**A**) PCA plot depicting differences in SCFA community structure among the three groups (FMT-Ctrl, FMT-CUMS, FMT-CDK). (**B**) Heatmap of fecal SCFA profiles across treatment groups, with red and blue indicating relatively high and low levels, respectively. (**C**–**E**) Quantification of significantly differential SCFAs, including acetic acid, propionic acid, and isobutyric acid. (**F**) Spearman correlation analysis was performed to assess the associations between eight SCFAs and key bacterial genera responsive to the combined treatment of DJZT and ketamine. Each square represents the correlation coefficient between SCFAs and microbial taxa. Blue indicates positive correlations, whereas red indicates negative correlations, with color intensity reflecting the strength of the correlation. Each bar represents the mean ± SEM (*n* = 3 mice per group). Statistical analysis was performed using one-way ANOVA followed by Tukey’s multiple comparisons test. PCA was performed based on the covariance matrix. * *p* < 0.05, ** *p* < 0.01. See Supplementary Tables S3-1 and S3-2 for detailed statistics.

Emerging evidence suggests that SCFAs, the main metabolites produced by bacterial fermentation of dietary fiber in the gastrointestinal tract, act as important signaling molecules in the pathophysiology and progression of various neuropsychiatric disorders, including major depressive disorder (MDD) [22,23]. Given that SCFAs represent a critical mechanistic link between gut microbiota and central nervous system function, we next sought to determine whether alterations in SCFA profiles underlie the antidepressant effects of microbiota from DJZT combined with ketamine. To this end, we performed targeted metabolomic analysis of fecal samples collected from mice with different FMT conditions to assess the diversity and composition of SCFAs. To explore overall differences in metabolic profiles among groups, principal component analysis (PCA) was performed. The first two principal components accounted for 62.5% and 21% of the total variance, respectively. PCA revealed clear separation between FMT-CUMS and FMT-Ctrl groups, whereas microbiota from DJZT-plus-ketamine-treated donors partially restored the metabolic profile toward that of FMT-Ctrl (Figure 3A). Replicates within each group clustered tightly, indicating high reproducibility. These results indicate that DJZT combined with ketamine markedly reshapes the metabolic profile compared with CUMS, consistent with the subsequent targeted analyses of key metabolites. Furthermore, eight SCFAs (acetic acid, propanoic acid, butanoic acid, isobutyric acid, valeric acid, isovaleric acid, hexanoic acid, and isohexanoic acid) were successfully identified in fecal samples (Figure 3B). As shown in Figure 3B–E, CUMS significantly reduced the levels of acetic acid, propanoic acid, and isobutyric acid, whereas no significant alterations were observed in the other five SCFAs. Strikingly, the reductions in these three SCFAs were markedly reversed by microbiota derived from DJZT combined with ketamine. These data suggest that SCFA remodeling may represent a critical metabolic link between gut microbiota alterations and the antidepressant effects of DJZT combined with ketamine.

To further explore the potential microbial contributors to SCFA alterations, we performed Spearman correlation analysis between SCFAs and key microbial taxa responsive to the combined treatment of DJZT and ketamine. The results showed that most SCFAs were significantly positively correlated with the abundances of *Alistipes, unclassified_f_Lachnospiraceae*, *Prevotellaceae_UCG-001*, *Alloprevotella*, and *Lachnospiraceae_NK4A136_group*, whereas acetic acid and hexanoic acid were negatively correlated with *norank_f__Muribaculaceae* and *Alloprevotella*, respectively (Figure 3F). Among these taxa, *Alistipes*, which was specifically enriched following the combined administration of DJZT and ketamine [21], exhibited the strongest positive correlations with acetic acid and isobutyric acid. Importantly, these two SCFAs were also markedly restored by the combined treatment in CUMS mice. Together, these findings suggest that *Alistipes* is positively associated with the production or regulation of acetic acid and isobutyric acid, which may be linked to the therapeutic effects of DJZT in combination with ketamine.

### 2.5. DJZT Combined with Ketamine Restores CUMS-Induced Reductions in Serum SCFAs

**Figure 4:**
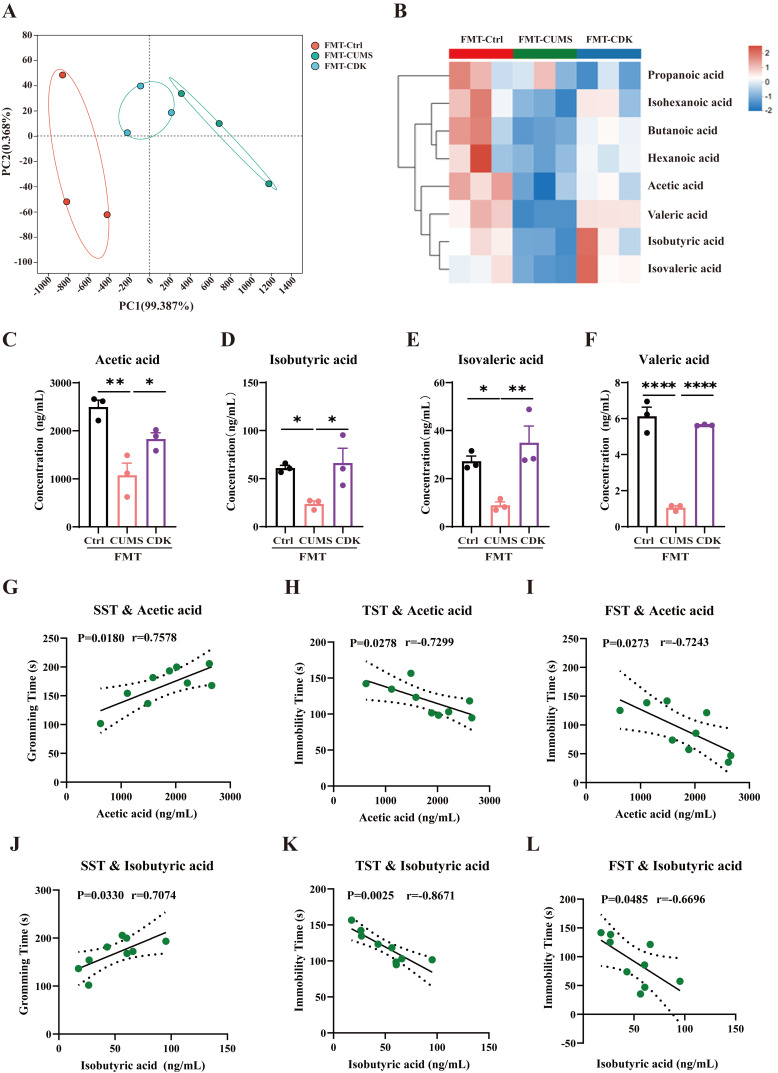
Serum SCFAs are altered by DJZT plus ketamine and are associated with behavioral outcomes. (**A**) PCA of serum SCFA profiles showing separation among the three groups. (**B**) Heatmap of relative serum SCFA levels across treatment groups, with red and blue indicating relatively high and low levels, respectively. (**C**–**F**) Quantification of serum SCFAs, including acetic acid, isobutyric acid, isovaleric acid, and valeric acid. (**G**–**I**) Spearman correlation analyses between serum acetic acid levels and behavioral performance (SST, TST, and FST). (**J**–**L**) Spearman correlation analyses between serum isobutyric acid levels and behavioral tests (SST, TST, and FST). Each bar represents the mean ± SEM (*n* = 3 mice per group). Statistical analysis was performed using one-way ANOVA followed by Tukey’s multiple comparisons test. PCA was performed based on the covariance matrix. * *p* < 0.05, ** *p* < 0.01, **** *p* < 0.0001. See Supplementary Tables S4-1 and S4-2 for detailed statistics.

To determine whether microbiota-derived SCFAs act as systemic mediators linking gut microbiota to the brain, we measured SCFA levels in serum. PCA of serum SCFA profiles revealed notable differences among the three groups (Figure 4A). Mice receiving CUMS microbiota were clearly separated from FMT-Ctrl mice, whereas the FMT-CDK group clustered closer to the FMT-Ctrl group, indicating that DJZT combined with ketamine partially restored the SCFA profile (Figure 4A). We further evaluated the composition of SCFAs in serum under various treatment, and eight SCFAs were successfully identified (Figure 4B). Compared with the FMT-Ctrl group, the relative levels of four SCFAs—acetic acid, isobutyric acid, isovaleric acid, and valeric acid—were significantly reduced in mice receiving the CUMS microbiota (Figure 4C–F). Importantly, microbiota from DJZT combined with ketamine markedly restored the levels of these four SCFAs (Figure 4C–F).

We next asked if SCFAs play a key role in stress-induced depressive-like behaviors. Notably, acetic acid and isobutyric acid exhibited the most consistent alterations across both fecal and serum samples, with similar trends observed following microbiota remodeling. In addition, these metabolites showed relatively robust group differences and have previously been implicated in gut–brain communication and neuroimmune regulation [24,25]. Therefore, acetic acid and isobutyric acid were selected for further analyses. To further evaluate the relationship between SCFAs and depressive-like behaviors, Spearman correlation analyses were performed. Acetic acid and isobutyric acid levels were positively correlated with grooming time in the SST and negatively correlated with immobility time in both the TST and FST (Figure 4G–L). These findings suggest that altered SCFA levels are associated with behavioral improvements following microbiota remodeling and support a potential association between SCFAs and antidepressant-like effects.

### 2.6. DJZT Combined with Ketamine Attenuates CUMS-Induced Neuroinflammation and Synaptic Dysfunction Through FFAR2

**Figure 5:**
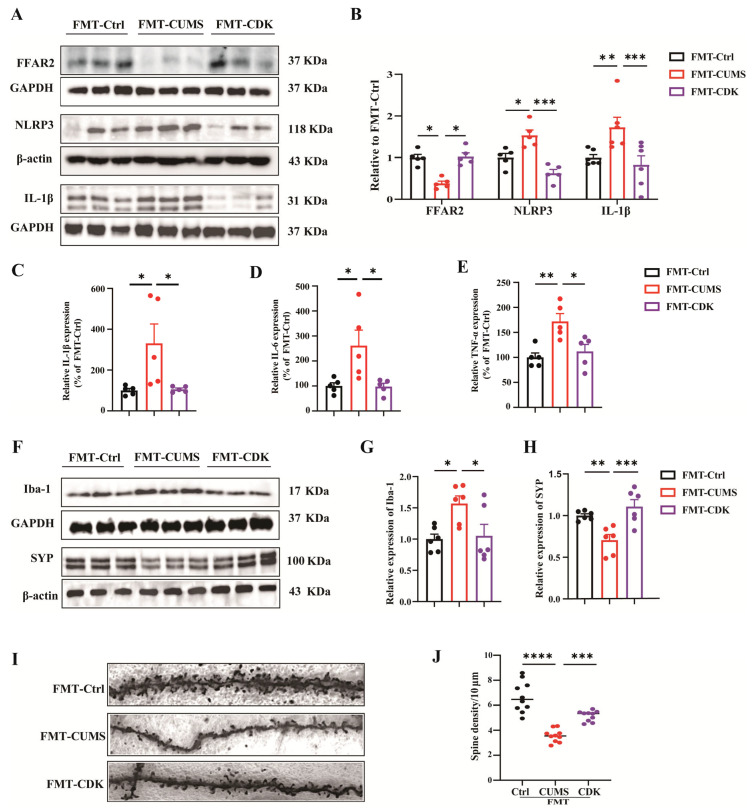
DJZT combined with ketamine attenuates CUMS-induced neuroinflammation and synaptic dysfunction through FFAR2. (**A**,**B**) Representative immunoblot images and quantitative analysis showing the expression of FFAR2, NLRP3, and IL-1β in the mPFC of mice from different treatment groups (FMT-Ctrl, FMT-CUMS, FMT-CDK). (**C**–**E**) qRT-PCR analysis of pro-inflammatory cytokines (IL-1β, IL-6, and TNF-α) in the mPFC. (**F**–**H**) Representative bands of Western blots and quantification of Iba-1 (microglial activation marker) and SYP (a marker of synaptic proteins) expression in the mPFC. (**I**,**J**) Golgi staining and quantification of dendritic spine density in the mPFC. Each bar represents the mean ± SEM (*n* = 5–10 per group). Statistical significance was determined by one- or two-way ANOVA followed by Tukey’s multiple comparisons test. * *p* < 0.05, ** *p* < 0.01, *** *p* < 0.001, **** *p* < 0.0001. See Supplementary Tables S5-1–S5-4 for detailed statistics.

Previous reports have demonstrated that SCFAs can be absorbed into the bloodstream and cross the blood–brain barrier (BBB) [26,27], where they exhibit anti-inflammatory properties, maintain microglial homeostasis, and promote synaptic number and function in the brain [28]. Mechanistically, it has been demonstrated that SCFAs regulate immune and inflammatory responses through activation of free fatty acid receptor 2 (FFAR2), also known as G-protein-coupled receptor 43 (GPR43) [29]. Here, we investigated the role of microbiota-derived SCFAs in FFAR2-mediated anti-inflammatory signaling. Western blot analysis revealed that microbiota from CUMS-treated mice significantly reduced FFAR2 expression, whereas microbiota transplantation from DJZT combined with ketamine-treated mice restored FFAR2 levels in the mPFC (Figure 5A,B). We further assessed whether FFAR2 negatively regulates downstream inflammatory mediators. Notably, microbiota derived from CUMS mice elevated the expression of NLRP3 and IL-1β, while these effects were markedly reversed following FMT from donors treated with the combination of DJZT and ketamine (Figure 5A,B). Together, these results imply that microbiota from DJZT combined with ketamine alleviates neuroinflammation in depression-like mice through the SCFAs–FFAR2–NLRP3–IL-1β signaling axis.

In agreement with these findings, qRT-PCR analysis showed that FMT-CDK significantly attenuated the elevated expression of pro-inflammatory cytokines, including IL-1β, IL-6, and TNF-α, induced by FMT-CUMS in the mPFC (Figure 5C–E). In parallel, the expression of the microglial activation marker Iba-1 was markedly reduced in FMT-CDK mice (Figure 5F,G), indicating suppressed microglial activation. Given that neuroinflammation and microglial activation are closely associated with synaptic dysfunction [9], we investigated synaptic integrity in response to combined DJZT and ketamine treatment. To comprehensively assess both molecular and structural aspects of synaptic plasticity, we measured the expression of the synaptic protein SYP and performed Golgi staining to evaluate dendritic spine morphology and density. Microbiota from DJZT combined with ketamine significantly increased SYP expression, enhanced dendritic spine density, and improved synaptic function (Figure 5F,H–J), suggesting a restoration of synaptic plasticity.

### 2.7. The Sustained Antidepressant Effect of DJZT Combined with Ketamine Is Abolished by an FFAR2 Antagonist

**Figure 6:**
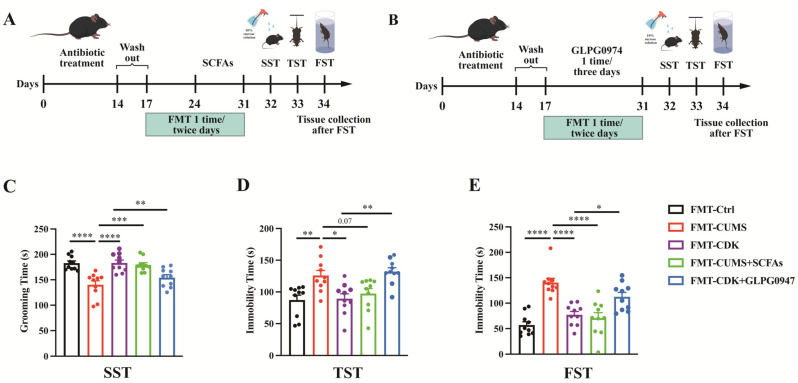
Microbiota-derived SCFAs exert antidepressant effects via FFAR2 activation. (**A**,**B**) Experimental timeline illustrating SCFA supplementation (the mixture of acetic acid and isobutyric acid) and administration of the FFAR2 antagonist GLPG0974, followed by behavioral assessments. The SST was conducted on day 32, followed by TST and FST. (**C**–**E**) Behavioral performance, including grooming time in the SST and immobility time in the TST and FST. SCFA supplementation significantly alleviated depressive-like behaviors, whereas pharmacological inhibition of FFAR2 by GLPG0974 abolished these effects. Each bar represents the mean ± SEM (*n* = 10 mice per group). Statistical analysis was performed using one-way ANOVA followed by Tukey’s multiple comparisons test. * *p* < 0.05, ** *p* < 0.01, *** *p* < 0.001, **** *p* < 0.0001. See Supplementary Tables S6-1 and S6-2 for detailed statistics.

Collectively, the above data indicate that DJZT synergizes with ketamine to sustain its antidepressant effects through modulation of gut-microbiota-derived SCFAs and the downstream FFAR2–NLRP3–IL-1β signaling pathway. To directly evaluate the functional contribution of microbiota-derived SCFAs, exogenous SCFAs were supplemented in antibiotic-cocktail-treated, pseudo-germ-free mice (Figure 6A). As shown in Figure 6C–E, compared with the FMT-CUMS group, mice receiving SCFA supplementation alone (FMT-CUMS + SCFAs) exhibited significant antidepressant-like behaviors, as evidenced by increased grooming time in the SST (Figure 6C) and reduced immobility time in both the TST (Figure 6D) and FST (Figure 6E), indicating that SCFA supplementation is sufficient to confer antidepressant-like effects.

To further determine whether these effects are mediated through FFAR2-dependent signaling, the selective FFAR2 antagonist GLPG0974 was administered (Figure 6B). Notably, mice in the FMT-CDK + GLPG0974 group failed to exhibit antidepressant-like behavioral improvements (Figure 6C–E), suggesting that blockade of FFAR2 abolishes the sustained antidepressant effects. Together, these results indicate that gut-microbiota-derived SCFAs act as key mediators of the sustained antidepressant synergy between DJZT and ketamine, with their effects being at least partially dependent on FFAR2 signaling.

### 2.8. Pharmacological Inhibition of FFAR2 Abolishes the Anti-Inflammatory and Synaptic Protective Effects of DJZT Combined with Ketamine

**Figure 7:**
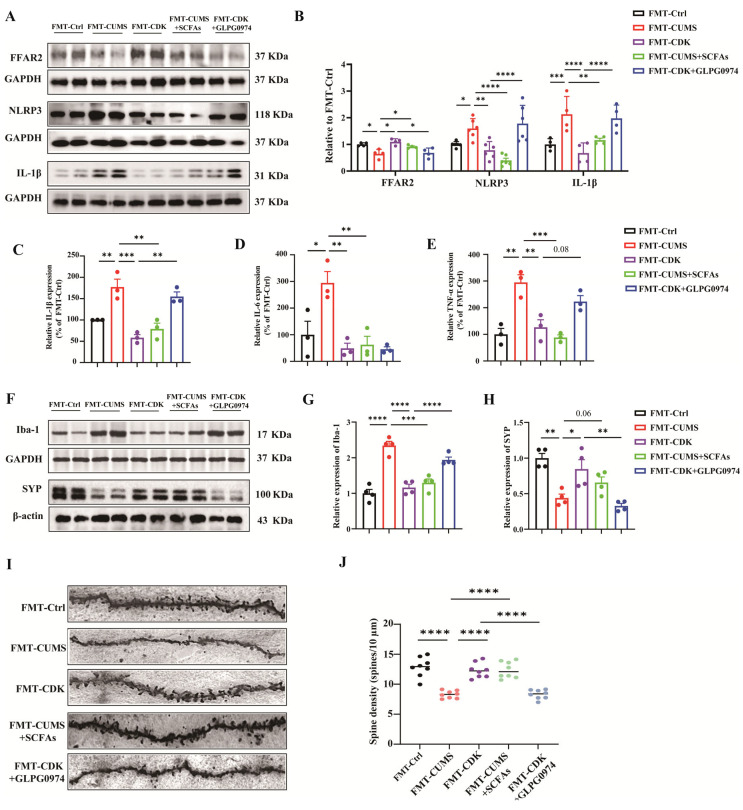
FFAR2 mediates the anti-inflammatory and synaptic protective effects of SCFAs and DJZT combined with ketamine-derived microbiota. (**A**,**B**) Representative immunoblot images and quantitative analysis showing the expression of FFAR2, NLRP3, and IL-1β in the mPFC of mice from different treatment groups (FMT-Ctrl, FMT-CUMS, FMT-CDK, FMT-CUMS+SCFAs, and FMT-CDK+GLPG0974) (*n* = 4–5 per group). (**C**–**E**) qRT-PCR analysis of pro-inflammatory cytokines (IL-1β, IL-6, and TNF-α) in the mPFC (*n* = 3 per group). (**F**–**H**) Representative images and quantification of Iba-1 and SYP expression in the mPFC (*n* = 4 per group). (**I**,**J**) Representative Golgi staining images and corresponding quantification of dendritic spine density in the mPFC across treatment groups (*n* = 8 per group). Each bar represents the mean ± SEM. Statistical significance was determined by one- or two-way ANOVA followed by Tukey’s multiple comparisons test. * *p* < 0.05, ** *p* < 0.01, *** *p* < 0.001, **** *p* < 0.0001. See Supplementary Tables S7-1–S7-4 for detailed statistics.

To further explore whether FFAR2 mediates the regulatory effects of microbiota-derived SCFAs on neuroinflammation and synaptic function, we examined FFAR2-associated signaling pathways using SCFA supplementation and pharmacological inhibition with GLPG0974. The results of immunoblot analysis showed that, compared with FMT-CUMS mice, SCFA supplementation (FMT-CUMS + SCFAs) significantly increased FFAR2 expression and suppressed the upregulation of NLRP3 and IL-1β induced by CUMS microbiota (Figure 7A,B), indicative of activation of FFAR2-dependent anti-inflammatory signaling. Consistently, qRT-PCR analysis revealed that SCFAs markedly reduced the elevated mRNA levels of IL-1β, IL-6, and TNF-α (Figure 7C–E). In contrast, pharmacological blockade of FFAR2 with GLPG0974 in FMT-CDK mice (FMT-CDK + GLPG0974) abolished the anti-inflammatory effects induced by CDK microbiota, as evidenced by the restoration of NLRP3 and IL-1β expression (Figure 7A,B) and increased pro-inflammatory cytokine levels (Figure 7C–E). Specifically, the suppressive effect on IL-1β was markedly reversed, and TNF-α showed a similar reversal trend, whereas IL-6 levels remained unchanged.

We next assessed microglial activation and synaptic plasticity in the mPFC. CUMS microbiota significantly increased Iba-1 expression and reduced SYP levels, whereas SCFA supplementation administered via oral gavage reversed these alterations (Figure 7F–H). Of note, GLPG0974 abolished the protective effects of CDK microbiota, leading to elevated Iba-1 expression and decreased SYP levels (Figure 7F–H). Similarly, Golgi staining demonstrated that both SCFA supplementation and CDK microbiota significantly increased dendritic spine density in the mPFC, while this effect was completely abolished by GLPG0974 in FMT-CDK recipient mice (Figure 7I,J).

## 3. Discussion

**Figure 8:**
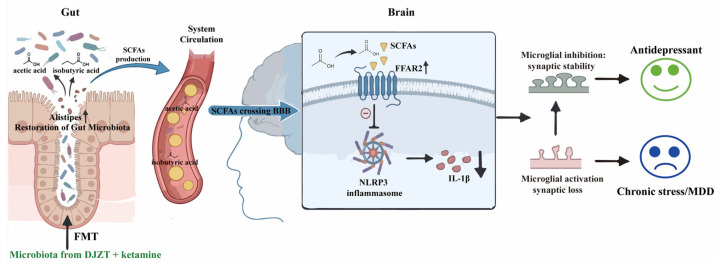
Schematic illustration of the microbiota–SCFA–FFAR2–NLRP3–IL-1β signaling pathway underlying the synergistic antidepressant effects of DJZT combined with ketamine. In chronic stress or MDD, gut microbiota dysbiosis has been observed, with reductions in SCFA-associated bacteria (e.g., *Alistipes*) and lower levels of key SCFAs, especially acetic acid and isobutyric acid. This is accompanied by downregulation of FFAR2 signaling and activation of neuroinflammatory pathways, including the NLRP3 inflammasome and IL-1β, in the mPFC, ultimately leading to microglia activation, synaptic dysfunction, and depressive-like behaviors. By contrast, DJZT combined with ketamine remodels the gut microbiota, restores SCFA production, and increases circulating levels of acetic acid and isobutyric acid. These metabolites activate FFAR2 signaling in the mPFC, suppress neuroinflammation, and preserve synaptic function. In conclusion, DJZT appears to synergize with ketamine by modulating a microbiota–SCFA–FFAR2–NLRP3–IL-1β signaling axis, which may link peripheral microbial metabolism to central neuroimmune regulation. Arrows indicate signaling direction, blunt-ended lines (⊣) indicate inhibition, upward arrows (↑) indicate activation or upregulation, and downward arrows (↓) indicate downregulation of expression.

In the present study, we systematically investigated how microbiota-derived signals along the gut–brain axis contribute to the combined therapeutic action of DJZT and ketamine. We found that microbiota derived from CUMS mice was sufficient to induce depressive-like behaviors, accompanied by reduced levels of SCFAs in both feces and serum, reduced FFAR2 expression in the mPFC, elevated pro-inflammatory cytokines, and synaptic deficits. It is noteworthy that these alterations were reversed by microbiota from mice treated with DJZT in combination with ketamine. Mechanistically, SCFA supplementation recapitulated these effects by activating FFAR2 and suppressing the NLRP3–IL-1β signaling pathway in the mPFC. Importantly, inhibition of FFAR2 by GLPG0974 abolished the antidepressant effects conferred by DJZT-plus-ketamine-derived microbiota and the associated anti-inflammatory and synaptic protective effects. Collectively, these findings identify a microbiota-dependent mechanism by which DJZT enhances ketamine efficacy and highlight the SCFA–FFAR2–NLRP3–IL-1β signaling axis as a key link between peripheral microbial metabolism and central synaptic and inflammatory regulation in the treatment of depression (Figure 8).

It is well established that gut microbiota play a critical role in the pathophysiology of depression [30,31,32,33]. FMT has emerged as a powerful approach to assess the causal role of microbiota in disease phenotypes [34,35,36]. In line with the existing literature [30,37], the current study confirms that FMT from CUMS mice was sufficient to induce depressive-like behaviors (Figure 2), supporting a causal role of dysbiotic gut microbiota. In contrast, FMT from donors treated with DJZT in combination with ketamine exerted antidepressant-like effects in recipient mice (Figure 2). These findings indicate that the gut microbiota serve as a vital mediator through which the synergistic effects of DJZT and ketamine are manifested. The successful translation of the antidepressant-like activity via FMT suggests that the therapeutic effects of the combined treatment are, at least in part, mediated by microbiota-dependent mechanisms.

SCFAs are key metabolites produced by the gut microbiota and defined as short-chain fatty acids containing fewer than six carbon atoms. They play a vital role in gut–brain axis communication by maintaining intestinal barrier integrity, modulating host immune responses, and influencing microglial function [23,38,39]. Reduced SCFA levels have been consistently reported in patients with depression [40,41], and recent meta-analyses further suggested that circulating SCFA profiles may serve as potential biomarkers of gut–brain axis dysfunction in MDD [42]. Consistent with these clinical observations, we found that multiple SCFAs were significantly reduced in both fecal and serum samples of CUMS mice. Notably, acetic acid and isobutyric acid were consistently increased and restored by the treatment with DJZT combined with ketamine (Figure 3 and Figure 4), suggesting their potential involvement in the therapeutic effects. Furthermore, correlation analyses revealed that acetic acid and isobutyric acid were significantly associated with behavioral outcomes, showing negative correlations with SST performance and positive correlations with immobility time in the TST and FST, supporting a potential link between specific SCFAs and depressive-like behaviors.

Acetic acid, one of the most abundant SCFAs, plays a key role in regulating host metabolic and immune homeostasis [43,44], while isobutyric acid, a branched-chain SCFA derived from microbial amino acid fermentation, has also been shown to be implicated in immune regulation and inflammatory modulation [45,46]. Although existing studies have predominantly focused on butyrate in depression, the roles of other SCFAs remain less well defined [47,48]. Moreover, previous reports have shown inconsistent alterations of SCFA levels between fecal and serum samples; a possible explanation for this difference is that it reflects variations in microbial production, host absorption, and systemic utilization [49]. However, a previous study by Wang and colleagues found that mice with depressive-like behaviors exhibited significantly reduced SCFA levels in both feces and serum, particularly acetic acid, which showed a positive correlation between fecal and circulating levels, along with increased inflammatory responses in the hippocampus [40]. Consistent with this, the parallel changes in fecal and circulating SCFAs observed in our study suggest a coordinated regulation of microbial metabolic activity and host SCFA utilization. This integrated regulation may be important for maintaining effective signaling in the gut–brain axis. Among the SCFAs examined, acetic acid and isobutyric acid were consistently altered, highlighting their potential role as key mediators in the modulation of the therapeutic effects of the combined treatment.

Emerging evidence from both animal and clinical studies suggests that SCFA levels are closely linked to specific microbial taxa. For instance, lower abundance of SCFA-producing bacteria has been associated with depressive symptoms in patients, including genera such as *Odoribacter, Alistipes, Anaerotruncus, Intestinimonas, Eubacterium,* and *Clostridiales* [50]. Aligned with this, our correlation analysis revealed that acetic acid and isobutyric acid were positively associated with *Alistipes*, a genus selectively enriched by DJZT combined with ketamine treatment (Figure 3). These findings suggest that *Alistipes* may contribute to the production or regulation of these SCFAs. Consistent with this notion, previous studies have shown that enrichment of *Alistipes* is associated with augmented SCFA production and improved gut microbial homeostasis [51,52,53]. Moreover, clinical evidence indicates that patients with major depressive disorder exhibit reduced levels of *Alistipes* compared to healthy controls [54]. Together, these data support a model in which DJZT combined with ketamine reshapes the gut microbiota, particularly by enriching *Alistipes*, potentially promoting the production of specific SCFAs such as acetic acid and isobutyric acid, which may be associated with its antidepressant-like effects. Nevertheless, further research is required to directly investigate the influence of *Alistipes* in mediating these effects, including its specific contribution to SCFA production and its functional involvement in the gut–brain axis.

It is well known that SCFAs exert potent anti-inflammatory effects through multiple mechanisms, including inhibition of histone deacetylases [55], which enhances protein acetylation and regulates gene transcription, as well as activation of G-protein-coupled receptors such as FFAR2. Among these pathways, FFAR2-mediated signaling has been increasingly recognized as a critical link between microbiota-derived metabolites and host immune regulation [56,57]. As reported previously, we observed that FFAR2 expression was significantly downregulated in the mPFC of CUMS mice and restored by treatment with a mixture of SCFAs (acetic acid and isobutyric acid) or with DJZT combined with ketamine (Figure 5). Supporting this, previous animal studies have shown that supplementation with SCFAs can improve gut microbiota composition and mitigate depressive-like behaviors in CUMS models [58]. Moreover, pharmacological inhibition of FFAR2 using the selective antagonist GLPG0974 abolished the antidepressant effects of microbiota from DJZT-plus-ketamine-treated donors and prevented the associated anti-inflammatory and synaptic protective effects (Figure 6 and Figure 7). These results imply that microbiota-derived SCFAs act as key upstream mediators linking peripheral microbial remodeling to central FFAR2-dependent signaling. Within this framework, DJZT and ketamine may exert complementary roles: DJZT primarily reshapes the gut microbial ecosystem to restore SCFA production, whereas ketamine acts on central neural circuits that are permissive to rapid antidepressant responses. The convergence of these processes at the level of SCFA–FFAR2 signaling provides a mechanistic basis for their synergistic interaction.

A growing body of evidence suggests that activation of FFAR2 can modulate the NLRP3 inflammasome pathway, thereby linking microbiota-derived signals to innate immune regulation [40,59,60]. The NLRP3 inflammasome, as a key mediator of innate immune activation, promotes release of pro-inflammatory cytokines such as IL-1β by microglial cells, thereby contributing to synaptic dysfunction and depressive-like behaviors [40,59,61]. It is worth noting that excessive inflammatory signaling can further drive microglial activation toward a synapse-engulfing phenotype, facilitating aberrant synaptic pruning and elimination [62]. In this context, microglia-mediated synaptic engulfment may counteract the synaptogenic effects of ketamine, leading to the removal of newly formed synapses. In the current study, we found that microbiota derived from CUMS mice significantly elevated NLRP3 and IL-1β expression in the mPFC, accompanied by microglial activation and synaptic deficits. Strikingly, these alterations were significantly attenuated in mice after colonization with microbiota from donors treated with DJZT combined with ketamine (Figure 5), indicating that the combined treatment effectively suppresses neuroinflammation, limits excessive microglia-mediated synaptic elimination, and preserves synaptic integrity.

In addition, NF-κB, a central transcriptional regulator of inflammatory responses, has been widely reported to control the expression of multiple pro-inflammatory genes, including key components of the NLRP3 inflammasome [63]. Although NF-κB signaling was not directly examined in the present study, previous studies, including our own work, have shown that DJZT can suppress NF-κB activation, raising the possibility that this pathway may contribute to its anti-inflammatory effects [21,64]. Therefore, it is likely that microbiota-derived SCFAs may modulate neuroinflammation through a FFAR2-dependent pathway that potentially involves NF-κB-mediated regulation of NLRP3 activation.

Importantly, the ability of microbiota from DJZT-plus-ketamine-treated donors to reproduce antidepressant-like effects in recipient mice provides evidence that these microbiota-derived signals are not merely correlative but contribute to the observed behavioral outcomes. This effect is likely mediated by the transfer of a reshaped microbial community with enhanced capacity for SCFA production, thereby restoring SCFA–FFAR2 signaling in recipient animals. Taken together, our findings support a model in which DJZT enhances the antidepressant efficacy of ketamine by remodeling the gut microbiota and restoring SCFA production, which in turn promotes FFAR2-dependent anti-inflammatory signaling in the brain. This microbiota–metabolite–receptor axis provides a mechanistic framework for the synergistic effects of DJZT and ketamine, as well as the transferable antidepressant-like effects observed after fecal microbiota transplantation.

The present study has several limitations. First, SCFA levels were not directly measured in the brain or cerebrospinal fluid, and the transport of SCFAs across the BBB was not assessed. Second, the untargeted metabolomics analyses were conducted with a relatively small sample size, which may reduce statistical power despite the overall consistency of the metabolic profiles and the support from downstream biological validations. Third, the cellular localization of FFAR2 in the mPFC was not determined. Although pharmacological inhibition indicated involvement of FFAR2 signaling, it remains unclear whether these effects occur primarily in microglia, neurons, or astrocytes. Additionally, while fecal microbiota transplantation demonstrated a functional role of the gut microbiota, specific causal bacterial strains were not identified, and this study primarily focused on SCFAs, leaving open the possibility that other microbiota-derived metabolites also contribute to the observed effects. Future studies employing larger metabolomics cohorts, brain metabolite profiling, strain-level microbiota analyses, and cell-specific approaches will be critical to further elucidate the mechanistic pathways linking the gut and brain.

## 4. Materials and Methods

### 4.1. Animals and Experimental Design

Adult male C57BL/6 mice (6–12 weeks old) were obtained from the Experimental Animal Center of Southern Medical University (Guangzhou, China). Animals were housed under standard laboratory conditions with a 12/12 h light–dark cycle and food and water *ad libitum*, except during the CUMS procedure, which included periods of water deprivation. Prior to stress exposure, mice were acclimated to the housing environment for one week. Animals subjected to stress were single-housed one day before DJZT administration. All procedures were approved by the Animal Care and Ethics Committee of Southern Medical University and were conducted in compliance with institutional guidelines.

Animals were randomly assigned to experimental groups using a computer-generated randomization list. Behavioral testing and data analysis were conducted by investigators blinded to group assignments. Animals with data points exceeding ±2 standard deviations from the group mean were excluded from analysis. Sample sizes were based on previous studies using similar experimental designs and endpoints [5,21].

### 4.2. Drug Administration

Dajianzhong Tang (DJZT; Japanese name: Dai-Kenchu-To; Tsumura Co., Tokyo, Japan) was administered orally (p.o.) at a dose of 300 mg/kg once daily. Ketamine (Sigma-Aldrich, St. Louis, MO, USA) was administered intraperitoneally (i.p.) at a dose of 10 mg/kg. All drugs were diluted in sterile 0.9% saline prior to administration. Beginning on day 14 of the CUMS procedure, mice received daily oral administration of DJZT or an equivalent volume of saline for 14 consecutive days until day 28. On day 21, mice in the ketamine group and the combined treatment group received a single dose of ketamine, while mice in the other groups were injected with an equivalent volume of saline. Behavioral tests were performed after completion of the CUMS procedure.

### 4.3. CUMS Procedure

Animals were exposed to one to two randomly scheduled and unpredictable stressors per day for 28 days according to a previously established method [21]. The same stressor was not applied on two consecutive days. The CUMS protocol included restraint stress, cage rotation, cage tilting, water deprivation, reversal of the light–dark cycle, rat feces, wet bedding, no bedding and white noise stress. Control animals were handled daily but were not exposed to any stressors.

### 4.4. Behavioral Studies

The detailed procedure and timeline for behavioral tests are shown in Figure 1A, Figure 2A, and Figure 6A. Mice were habituated to the testing room for 30 min prior to each behavioral assessment. All behavioral experiments were conducted between 10 a.m. and 3 p.m. Four behavioral tests, including SST, NSFT, TST, and FST, were measured as reported previously [5,21]. For SST, the mice were sprayed with 10% sucrose solution on their head or back and then placed into a new clean cage. The grooming time within a 5 min observation period was counted. In NSFT, mice were food-deprived for 16–18 h and placed in an open-field arena with a food pellet positioned at the center. The latency to begin feeding was recorded, followed by measurement of home cage food intake for 20 min. In TST, mice were suspended by the tail, and immobility time was recorded during the last 4 min of a 6 min session. In FST, mice were placed in a glass cylinder filled with water (18 cm depth, 24 ± 1 °C) and allowed to swim for 6 min. Immobility time was recorded during the last 4 min of the test.

### 4.5. Western Blotting

Synaptic and cytoplasmic proteins were extracted from mPFC synaptosomal and cytoplasmic fractions using RIPA lysis buffer supplemented with protease and phosphatase inhibitor cocktails. The primary antibodies were used in western blots: rabbit anti-synaptophysin (SYP) (1:1000, cell signaling technology, Danvers, MA, USA), rabbit anti-Iba-1 (1:1000, Cell Signaling, Danvers, MA, USA), mouse anti–interleukin-1β (IL-1β) (1:500, HuaAn, Hangzhou, China), rabbit anti–NLR family pyrin domain containing 3 (NLRP3) (1:500–1:1000, Affinity Biosciences, Cincinnati, OH, USA), rabbit anti–free fatty acid receptor 2 (FFAR2) (1:500, Proteintech, Rosemont, IL, USA), rabbit anti-GAPDH (1:30,000, HuaAn, China), and mouse anti-β-actin (1:30,000, HuaAn, China). Immunoreactive bands were visualized and quantified using ImageJ Fiji software. Protein expression levels were normalized to GAPDH or β-actin as internal loading controls.

### 4.6. Golgi Staining

Mice were deeply anesthetized and decapitated, and the brains were rapidly removed and immersed in Golgi–Cox solution (prepared by mixing 5% potassium dichromate, 5% mercuric chloride, and 5% potassium chromate at a ratio of 5:5:4). Brain tissues were impregnated in the dark at room temperature for 14 days. After impregnation, brains were transferred to the washing solution provided in the FD Rapid GolgiStain™ Kit (FD NeuroTechnologies, Columbia, MD, USA) and stored in the dark at room temperature for at least 72 h. The washing solution was replaced once after the first 24 h. Coronal sections (100–200 μm thickness) were prepared using a cryostat (Thermo Fisher, Waltham, MA, USA) at −20 °C to −22 °C. Sections were mounted directly onto gelatin-coated glass slides and air-dried in the dark at room temperature. Subsequently, sections were stained with the working solution from the FD Rapid GolgiStain™ Kit (reagents D and E mixed at a ratio of 1:1:2 with distilled water) for 10 min, followed by coverslipping with glycerol-based mounting medium. Images were acquired using a Zeiss upright fluorescence microscope (Zeiss, Oberkochen, Germany). For each mouse, dendritic segments (≥20 μm in length) within the mPFC were consecutively imaged at 80× magnification. Dendritic spine numbers were quantified using the NeuronJ plugin in ImageJ software, and spine density was calculated as the number of spines per 10 μm of dendritic length.

### 4.7. Preparation of Fecal Suspension

Donor mice were derived from the control group, the CUMS group, and the combined treatment group (CUMS + DJZT + ketamine). Fresh fecal pellets were collected under sterile conditions, immediately placed on ice, and stored at −80 °C within 30 min until further use. For preparation of fecal suspensions, donor feces were resuspended at 0.9% sterile saline (e.g., 200 mg feces in 2 mL sterile saline). Samples were thoroughly homogenized until no visible large particles remained, vortexed, and filtered through a 0.5 mm nylon mesh to remove debris. The filtrate was centrifuged at 600× *g* for 5 min at 4 °C, and the supernatant was collected as the fecal bacterial fluid. Sterile glycerol was added to a final concentration of 10% as a cryoprotectant. The suspension was aliquoted and stored at −80 °C until transplantation.

### 4.8. Establishment of Pseudo-Germ-Free Mice

Adult male C57BL/6J mice (6–8 weeks old) were used for the establishment of pseudo-germ-free mice. To deplete the endogenous gut microbiota, mice were administered a broad-spectrum antibiotic cocktail via drinking water for 14 days. The antibiotic cocktail consisted of ampicillin (1 g/L), neomycin sulfate (1 g/L), vancomycin (0.5 g/L), and metronidazole (1 g/L). Following antibiotic cocktail treatment, mice underwent a 3-day washout period without antibiotics to eliminate residual drugs from the gastrointestinal tract prior to subsequent experiments.

### 4.9. Fecal Microbiota Transplantation

Pseudo-germ-free C57BL/6J recipient mice were randomly assigned to three groups: the FMT-Ctrl group (receiving fecal microbiota from control donors), the FMT-CUMS group (receiving fecal microbiota from CUMS donors), and the FMT-CDK group (receiving fecal microbiota from donors treated with DJZT and ketamine). FMT was performed via oral gavage (p.o.). Each mouse received 200 μL of fecal suspension per administration once every 2 days for 14 consecutive days (a total of seven administrations). Behavioral assessments were conducted on the day following the final gavage.

### 4.10. Targeted Metabolomics

#### 4.10.1. Fecal Samples

SCFAs were extracted from fecal samples (25 mg per sample) using a methanol-based extraction solution containing 10 μg/mL 2-ethylbutyric acid as an internal standard. Gas chromatography–mass spectrometry (GC–MS) analysis was subsequently performed using an Agilent 8890B gas chromatograph coupled with an Agilent 5977B mass selective detector equipped with an inert electron impact (EI) ionization source (Agilent, Santa Clara, CA, USA). The ionization energy was set at 70 eV. All analyses were conducted at Majorbio Bio-Pharm Technology Co., Ltd. (Shanghai, China). Data acquisition was carried out in full-scan mode. Compounds were identified and quantified using MassHunter Quantitative Analysis software (Agilent, USA; version 10.0.707.0). The resulting concentration data matrix was uploaded to the Majorbio Cloud Platform (cloud.majorbio.com) for downstream analysis. PCA was performed using the R package ropls (version 1.6.2).

#### 4.10.2. Serum Samples

Serum samples (50 μL) were mixed with 100 μL acetonitrile, followed by sonication for 30 min and centrifugation at 13,000× *g* for 15 min at 4 °C. Subsequently, 20 μL of the supernatant was derivatized with 20 μL of 200 mM 3-nitrophenylhydrazine hydrochloride (3-NPH·HCl) and 20 μL of 120 mM EDC·HCl (containing 6% pyridine) at 40 °C for 30 min. The reaction mixture was then diluted to 750 μL with 50% aqueous acetonitrile prior to analysis. LC–MS/MS analysis was performed using an ExionLC AD system coupled with a QTRAP^®^ 6500+ mass spectrometer (Sciex, Framingham, MA, USA) equipped with an electrospray ionization (ESI) source operating in negative mode at Majorbio Bio-Pharm Technology Co., Ltd. (Shanghai, China). Raw data were processed using Sciex software OS. (Sciex, USA), with automatic peak identification and integration followed by manual inspection. Metabolite concentrations were quantified based on standard calibration curves. The resulting data matrix was uploaded to the Majorbio Cloud Platform (cloud.majorbio.com) for further analysis. PCA was conducted using the R package ropls (version 1.6.2), and model stability was evaluated by seven-fold cross-validation.

### 4.11. SCFA Supplementation

Mice in the FMT-CUMS group were used as recipients for SCFA supplementation. Sodium acetate (>98%, reagent grade; Beyotime, China), and sodium isobutyrate (97%; Beyotime, Shanghai, China) were dissolved in sterile water to prepare a mixed solution with final concentrations of 67.5 mM sodium acetate and 40 mM sodium isobutyrate. The SCFA mixture was administered daily for 14 days. Control mice received an equivalent volume of sterile water.

### 4.12. Administration of GLPG0974

Mice in the FMT-CDK group were further treated with the FFAR2 antagonist GLPG0974 to block FFAR2 signaling. GLPG0974 (5 mg; purity 98%; Beyotime, China) was dissolved in 50 μL DMSO and vortexed until fully dissolved to obtain a stock solution at a concentration of 100 mg/mL. A total of 25 μL of the stock solution was diluted in 25 mL sterile saline to prepare a working solution with a final concentration of 0.1 mg/mL. Recipient mice were treated via oral gavage every three days (on days 1, 4, 7, 10, and 13). The administration volume was 0.1 mL per 10 g body weight, corresponding to the dose of 0.98 mg/kg per administration. Control mice received an equal volume of saline containing 0.1% DMSO.

### 4.13. Quantitative Real-Time Polymerase Chain Reaction (qRT-PCR)

The mPFC tissues were collected for qRT-PCR analysis. Total RNA was extracted from tissue samples using a Total RNA Extraction Kit (Vazyme, Nanjing, China) according to the manufacturer’s instructions. RNA concentration and purity were determined using a DeNovix DS-11 Plus spectrophotometer. All samples exhibited A260/A280 ratios between 1.8 and 2.0. For cDNA synthesis, 1 μg of total RNA was reverse transcribed using the HiScript II Q RT SuperMix for qPCR kit (Vazyme, China). Quantitative PCR was performed using ChamQ Universal SYBR qPCR Master Mix (Vazyme, China) on an Applied Biosystems QuantStudio 3 Real-Time PCR system. GAPDH was used as the internal reference gene. Relative gene expression levels were calculated using the 2^^−ΔΔCt^ method, where ΔCt = Ct (target gene) − Ct (GAPDH), and ΔΔCt = ΔCt (experimental group) − ΔCt (control group). Results were expressed as fold changes of the target gene normalized to GAPDH.

### 4.14. Statistical Analysis

Statistical analyses were performed using IBM SPSS Statistics version 23. Data distribution was assessed for normality using the Shapiro–Wilk test prior to statistical analysis. For datasets showing normal distribution, parametric tests were applied; one-way or two-way ANOVA followed by Tukey’s post hoc test. Post hoc analyses were performed only when the omnibus ANOVA was statistically significant. For fecal and serum samples involved in SCFA-targeted metabolomic analysis, PCA was performed based on the covariance matrix. Results are presented as the mean ± standard error of the mean (SEM). A value of *p* < 0.05 was considered statistically significant. All bar graphs were generated using GraphPad Prism 9 software.

## 5. Conclusions

In summary, our findings support the involvement of a microbiota–metabolite–receptor axis in the synergistic antidepressant-like effects of DJZT and ketamine in a preclinical mouse model. Specifically, DJZT combined with ketamine remodeled the gut microbiota and was associated with the restoration of key SCFAs, particularly acetic acid and isobutyric acid, alongside FFAR2-dependent regulation of neuroinflammatory signaling in the mPFC. These changes were accompanied by reduced NLRP3–IL-1β signaling, preservation of synaptic integrity, and improvements in depressive-like behaviors. Collectively, these findings provide mechanistic insights into the potential link between microbiota-derived metabolites and central neural function and suggest that the gut microbiota–SCFA–FFAR2 signaling pathway may represent a promising target for future antidepressant research, which will require further validation in translational and clinical studies.

## Data Availability

The original contributions presented in this study are included in the article/Supplementary Material. Further inquiries can be directed to the corresponding author(s).

## Supplementary Materials

The following supporting information can be downloaded at: https://www.mdpi.com/article/10.3390/ph19060877/s1, Figure S1: Identification of major components of DJZT extract by UPLC-OE-MS in positive ion mode; Figure S2: Identification of major components of DJZT extract by UPLC-OE-MS in negative ion mode. Table S1-1: Behavioral parameters in each group (Mean ± SEM); Table S1-2: Comparison of behavioral parameters among five groups (*p*-values); Table S2-1: Behavioral parameters in each group (Mean ± SEM); Table S2-2: Comparison of behavioral parameters among three groups (*p*-values); Table S3-1: Fecal SCFAs parameters in each group (Mean ± SEM); Table S3-2: Comparison of fecal SCFAs levels among three groups (*p*-values); Table S4-1: Serum SCFAs parameters in each group (Mean ± SEM); Table S4-2: Comparison of serum SCFAs levels among three groups (*p*-values); Table S5-1: Relative expression of proteins in the mPFC for each group (Mean±SEM); Table S5-2: Relative expression of mRNA and spine density in the mPFC for each group (Mean ± SEM); Table S5-3: Comparison of relative expression of proteins among three groups (*p*-values); Table S5-4: Comparison of relative expression of mRNA and spine density among three groups (*p*-values); Table S6-1: Behavioral parameters in each group (Mean ± SEM); Table S6-2: Comparison of behavioral parameters among five groups (*p*-values); Table S7-1: Relative expression of proteins in the mPFC for each group (Mean ± SEM); Table S7-2: Relative expression of mRNA and spine density in the mPFC for each group (Mean ± SEM); Table S7-3: Comparison of relative expression of proteins among five groups (*p*-values); Table S7-4: Comparison of relative expression of mRNA and spine density among five groups (*p*-values).

## Abbreviations

BBB	Blood–brain barrier
CUMS	Chronic unpredictable mild stress
DJZT	Dajianzhong Decoction
FFAR2	Free fatty acid receptor 2
FMT	Fecal microbiota transplantation
FST	Forced swim test
GPR43	G-protein-coupled receptor 43
IL-1β	Interleukin-1β
NLRP3	NLR family pyrin domain containing 3
NSFT	Novelty-suppressed feeding test
MDD	Major depressive disorder
mPFC	Medial prefrontal cortex
PCA	Principal component analysis
SCFAs	Short-chain fatty acids
SSRIs	Selective serotonin reuptake inhibitors
SST	Sucrose splash test
TCM	Traditional Chinese medicine
TST	Tail suspension test
UHPLC–OE–MS	Ultra-high performance liquid chromatography coupled with an Orbitrap Exploris 120 mass spectrometer
