# Supplementary material for: Microbiota-Derived SCFAs Mediate the Synergistic Antidepressant Effects of Dajianzhong Decoction and Ketamine via FFAR2-NLRP3-IL-1β Signaling
Source: Pharmaceuticals (Basel). 2026 May 31;19(6):877. doi: 10.3390/ph19060877 (PMC13304611; doi:10.3390/ph19060877)
Supplement: Supplementary file 1 [file pharmaceuticals-19-00877-s001.zip › pharmaceuticals-4283468-supplementary.pdf]

Supplementary Materials and Methods

UHPLC-OE-MS analysis

Dajianzhong Tang (DJZT; Japanese name: Dai-Kenchu-To; Tsumura Co., Japan) consists of *Zingiber officinale* Rose, *Zanthoxyli bungeanum* Maxim., *Panax ginseng* C. A. Mey, and maltose. The powder (50 ± 2 mg) were lyophilized and extracted with 500 μL of pre-chilled methanol/acetonitrile/water (2:2:1, v/v/v) containing deuterated internal standards. After vortexing (30 s), homogenization (35 Hz, 240 s), and sonication (5 min, 4 °C) repeated three times, samples were incubated at −40 °C for 30 min to precipitate proteins and centrifuged at 12,000 rpm for 15 min at 4 °C. The supernatants were collected, re-centrifuged under the same conditions, and transferred to glass vials for analysis. Quality control samples were prepared by pooling equal aliquots of all samples. LC–MS/MS analysis was performed using a Vanquish UHPLC system (Thermo Fisher, USA) coupled to an Orbitrap Exploris 120 mass spectrometer, with separation on a Phenomenex Kinetex C18 column (2.1 × 100 mm, 2.6 μm). The mobile phases consisted of 0.01% acetic acid in water (A) and isopropanol/acetonitrile (1:1, v/v) (B), with an injection volume of 2 μL and autosampler temperature maintained at 4 °C. Data were acquired in data-dependent acquisition mode using Xcalibur software, with ESI source parameters set as follows: sheath gas, 50 Arb; auxiliary gas, 15 Arb; capillary temperature, 320 °C; spray voltage, +3.8 kV (positive) or −3.4 kV (negative); full MS and MS/MS resolutions of 60,000 and 15,000, respectively; and stepped normalized collision energy of 20/30/40. Raw data were converted to mzXML format using ProteoWizard and processed using an in-house R-based pipeline built on XCMS for peak detection, alignment, and integration, with metabolite identification performed using R packages in combination with BiotreeDB (V3.0).

A

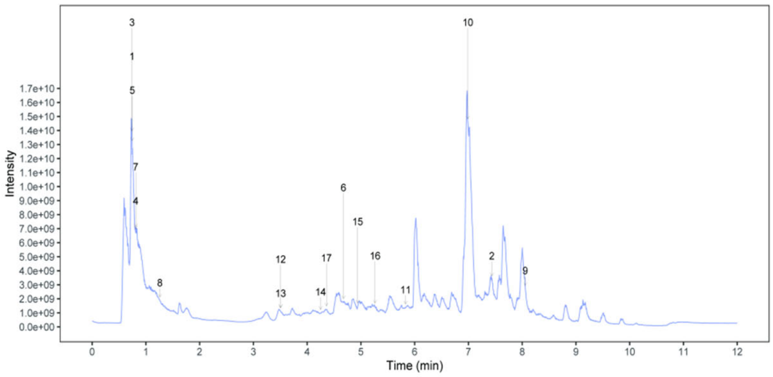

B

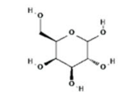

1
Galactose

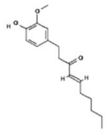

2
Shogaol

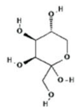

3
Tagatose

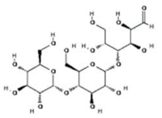

4
Maltotriose

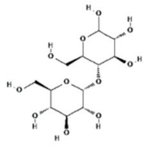

5
Maltose

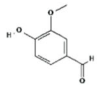

6
Vanillin

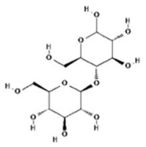

7
D-(+)-Cellobiose

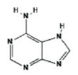

8
Adenine

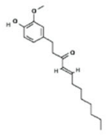

9
[8]-Shogaol

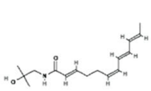

10
Hydroxy-α-sanshool

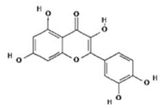

11
Quercetin

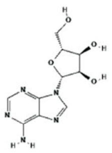

12
Adenosine

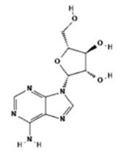

13
Vidarabine

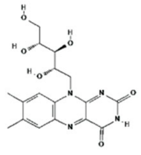

14
Riboflavin

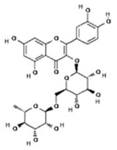

15
Rutin

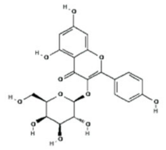

16
Kaempferol-3-O-galactoside

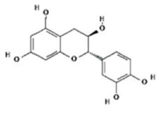

17
(-)-Catechin

Figure S1. Identification of major components of DJZT extract by UPLC-OE-MS in positive ion mode. (A) Total ion chromatogram (TIC) of the DJZT extract, in which 17 peaks were identified as representative characteristic compounds in positive ion mode. (B) Chemical structures of the identified 17 compounds.

A

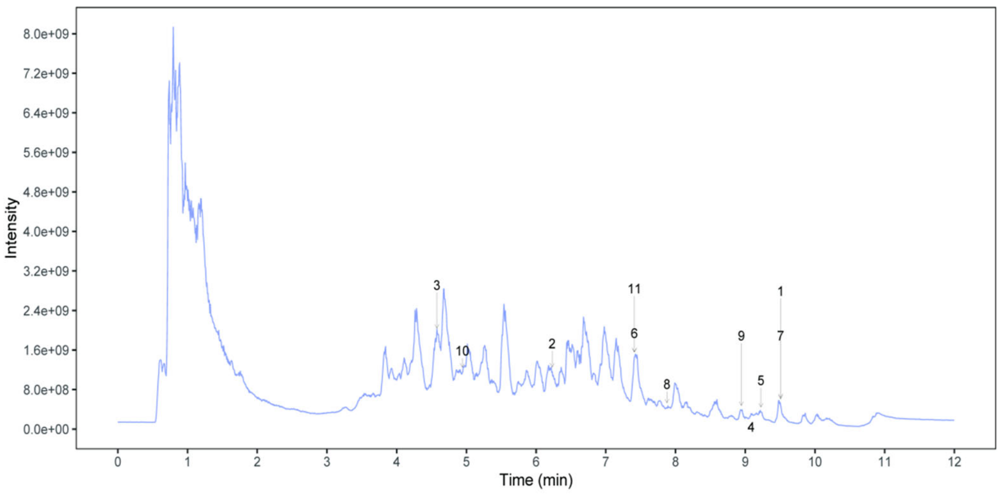

B

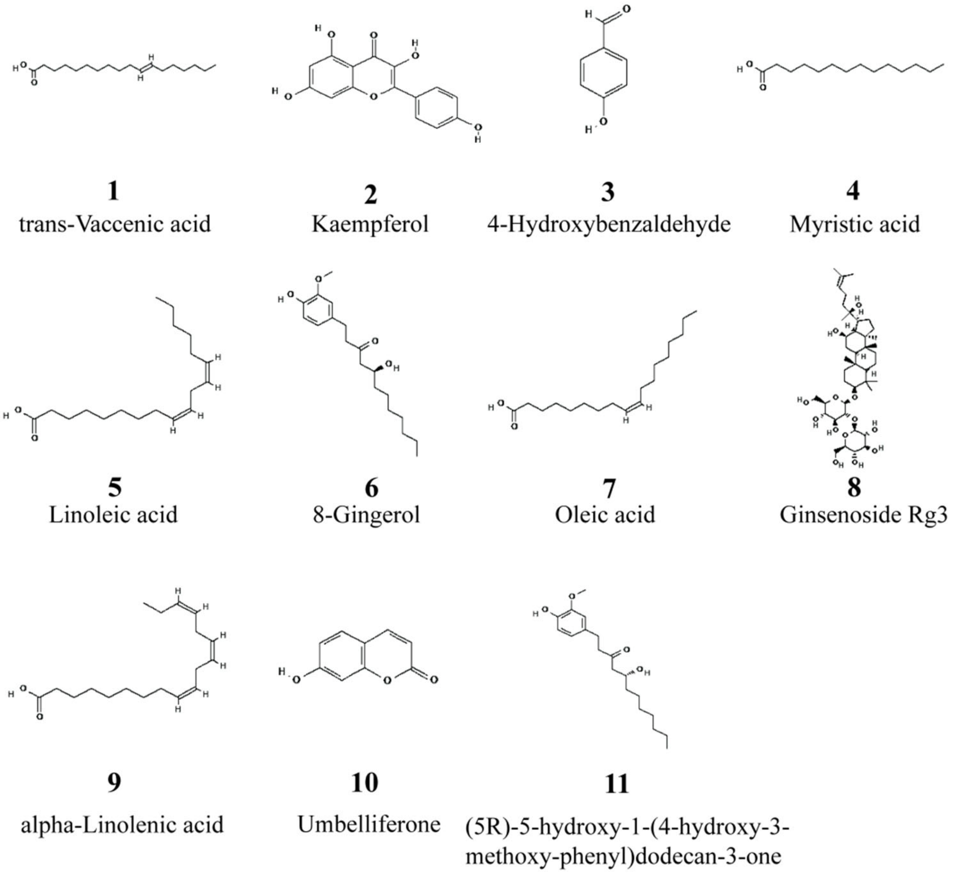

Figure S2. Identification of major components of DJZT extract by UPLC-OE-MS in negative ion mode. (A) TIC of the DJZT extract, in which 11 peaks were identified as representative characteristic compounds in negative ion mode. (B) Chemical structures of the identified 11 compounds.

Table S1-1. Behavioral parameters in each group (Mean ± SEM)

Group	SST (s)	NSFT (s)	TST (s)	FST (s)
Ctrl	171.7±5.495	107.0±20.34	74.97±8.078	73.12±9.198
CUMS	117.3±7.439	214.0±38.32	124.2±7.154	128.6±9.511
CUMS+DJZT	137.0±4.786	211.2±18.34	113.1±7.575	118.8±9.820
CUMS+KET	130.9±6.913	230.9±29.81	108.1±6.780	135.4±5.786
CUMS+DJZT+KET	156.4±6.827	208.3±15.81	94.65±5.650	80.30±8.802

Table S1-2. Comparison of behavioral parameters among five groups (P-values)

Group comparison	SST	NSFT	TST	FST
CUMS vs. Ctrl	<0.0001	0.0173	<0.0001	0.0001
CUMS vs. CUMS+DJZT	0.1046	0.9999	0.6477	0.8473
CUMS vs. CUMS+KET	0.3701	0.9735	0.3299	0.9503
CUMS vs. CUMS+DJZT+KET	0.0002	0.9997	0.0185	0.0010

Table S2-1. Behavioral parameters in each group (Mean ± SEM)

Group	SST (s)	TST (s)	FST (s)
FMT-Ctrl	182.9±4.222	94.40±5.566	90.15±11.07
FMT-CUMS	152.7±4.137	122.2±6.883	122.5±6.804
FMT-CDK	172.9±4.526	79.62±5.250	89.97±9.505

Table S2-2. Comparison of behavioral parameters among three groups (P-values)

Group comparison	SST	TST	FST
FMT-CUMS vs. FMT-Ctrl	<0.0001	0.0042	0.0332
FMT-CUMS vs. FMT-CDK	0.0044	<0.0001	0.0365

Table S3-1. Fecal SCFAs parameters in each group (Mean ± SEM)

Group	Acetic acid (μg/mg)	Propanoic acid (μg/mg)	Isobutyric acid (μg/mg)
FMT-Ctrl	2.654±0.4358	0.6382±0.09432	0.04169±0.005096
FMT-CUMS	0.7782±0.03915	0.2490±0.03765	0.01629±0.004527
FMT-CDK	2.333±0.4307	0.7373±0.04535	0.04197±0.003708

Table S3-2. Comparison of fecal SCFAs levels among three groups (P-values)

Group comparison	Acetic acid	Propanoic acid	Isobutyric acid
FMT-CUMS vs. FMT-Ctrl	0.0170	0.0010	0.0166
FMT-CUMS vs. FMT-CDK	0.0370	0.0031	0.0158

Table S4-1. Serum SCFAs parameters in each group (Mean ± SEM)

Group	Acetic acid (ng/mg)	Isobutyric acid (ng/mg)	Isovaleric acid (ng/mg)	valeric acid (ng/mg)
FMT-Ctrl	2497±141.6	61.08±2.687	27.23±2.154	6.127±0.5073
FMT-CUMS	1075±250.4	23.66±3.094	8.907±1.416	1.059±0.1006
FMT-CDK	1830±128.6	66.23±15.37	34.93±6.955	5.634±0.02260

Table S4-2. Comparison of serum SCFAs levels among three groups (P-values)

Group comparison	Acetic acid	Isobutyric acid	Isovaleric acid	valeric acid
FMT-CUMS vs. FMT-Ctrl	0.0027	0.0489	0.0406	<0.0001
FMT-CUMS vs. FMT-CDK	0.0458	0.0297	0.0091	<0.0001

Table S5-1. Relative expression of proteins in the mPFC for each group (Mean ± SEM)

Group	FFAR2	NLRP3	IL-1β	SYP	Iba-1
FMT-Ctrl	1.000±0.07780	1.000±0.1011	1.000±0.07340	1.000±0.02222	1.000±0.07883
FMT-CUMS	0.3814±0.0555	1.533±0.1308	1.727±0.2425	0.7055±0.06706	1.569±0.1212
FMT-CDK	1.023±0.09220	0.6248±0.09081	0.8263±0.2167	1.108±0.08361	1.053±0.1828

Table S5-2. Relative expression of mRNA and spine density in the mPFC for each group (Mean ± SEM)

Group	IL-1β	IL-6	TNF-α	Golgi
FMT-Ctrl	1.000±0.09782	1.000±0.1288	1.000±0.08937	6.679±0.3897
FMT-CUMS	3.305±0.9525	2.613±0.6205	1.721±0.1542	3.562±0.1611
FMT-CDK	1.058±0.06253	0.9783±0.1316	1.120±0.1404	5.154±0.1305

Table S5-3. Comparison of relative expression of proteins among three groups (P-values)

Group comparison	P value of FFAR2	P value of NLRP3	P value of IL-1β	P value of SYP	P value of Iba-1
FMT-CUMS vs. FMT-Ctrl	0.0159	0.0423	0.0016	0.0092	0.0169
FMT-CUMS vs. FMT-CDK	0.0121	0.0003	0.0001	0.0008	0.0295

Table S5-4. Comparison of relative expression of mRNA and spine density among three groups (P-values)

Group comparison	P value of IL-1β	P value of IL-6	P value of TNF-α	P value of Golgi
FMT-CUMS vs. FMT-Ctrl	0.0226	0.0184	0.0040	<0.0001
FMT-CUMS vs. FMT-CDK	0.0258	0.0171	0.0130	0.0003

Table S6-1. Behavioral parameters in each group (Mean ± SEM)

Group	Mean±SEM of SST	Mean±SEM of TST	Mean±SEM of FST
FMT-Ctrl	182.9±4.357	87.17±7.804	56.92±6.594
FMT-CUMS	140.3±7.603	125.9±8.139	140.5±8.310
FMT-CDK	183.2±5.616	89.36±7.712	77.17±6.561
FMT-CUMS+SCFAs	179.3±4.236	97.41±7.992	70.65±10.71
FMT-CDK+GLPG0974	154.3±5.782	132.0±6.137	112.6±8.510

Table S6-2. Comparison of behavioral parameters among five groups (P-values)

Group comparison	P value of SST	P value of TST	P value of FST
FMT-Ctrl vs. FMT-CUMS	<0.0001	0.0067	<0.0001
FMT-CUMS vs. FMT-CDK	<0.0001	0.0118	<0.0001
FMT-CUMS vs. FMT-CUMS+SCFAs	0.0001	0.0783	<0.0001
FMT-CDK vs. FMT-CDK+GLPG0974	0.0065	0.0022	0.0317

Table S7-1. Relative expression of proteins in the mPFC for each group (Mean ± SEM)

Group	FFAR2	NLRP3	IL-1β	SYP	Iba-1
FMT-Ctrl	1.000±0.0316	1.000±0.0428	1.000±0.107	1.000±0.067	1.000±0.1069
FMT-CUMS	0.649±0.0836	1.595±0.151	2.131±0.334	0.4406±0.053	2.131±0.334
FMT-CDK	1.099±0.0559	0.782±0.134	0.816±0.284	0.847±0.132	0.816±0.284
FMT-CUMS+SCFAs	0.916±0.0290	0.401±0.080	1.07±0.143	0.657±0.079	1.068±0.143
FMT-CDK+GLPG0974	0.684±0.0906	1.782±0.280	1.978±0.248	0.328±0.035	1.978±0.248

Table S7-2. Relative expression of mRNA and spine density in the mPFC for each group (Mean ± SEM)

Group	IL-1β	IL-6	TNF-α	Golgi
FMT-Ctrl	1.000±0.0014	1.000±0.5093	1.000±0.221	12.88±0.576
FMT-CUMS	1.774±0.1836	2.945±0.4256	2.948±0.291	8.283±0.220
FMT-CDK	0.585±0.0719	0.4921±0.194	1.264±0.284	12.31±0.442
FMT-CUMS+SCFAs	0.7914±0.134	0.6273±0.627	0.883±0.098	12.39±0.489
FMT-CDK+GLPG0974	1.549±0.1077	0.457±0.091	2.232±0.220	8.302±0.269

Table S7-3. Comparison of relative expression of proteins among five groups (P-values)

Group comparison	FFAR2	NLRP3	IL-1β	SYP	Iba-1
FMT-Ctrl vs. FMT-CUMS	0.0104	0.0372	<0.0001	0.0014	0.0307
FMT-CUMS vs. FMT-CDK	0.0013	0.0016	<0.0001	0.0195	0.0108
FMT-CUMS vs. FMT-CUMS+SCFAs	0.0622	<0.0001	<0.0001	0.0635	0.0448
FMT-CDK vs. FMT-CDK+GLPG0974	0.0026	<0.0001	0.0317	0.0020	0.0259

Table S7-4. Comparison of relative expression of mRNA and spine density among five groups (P-values)

Group comparison	IL-1β	IL-6	TNF-α	Golgi
FMT-Ctrl vs. FMT-CUMS	0.0060	0.0162	0.0011	<0.0001
FMT-CUMS vs. FMT-CDK	0.0002	0.0034	0.0033	<0.0001
FMT-CUMS vs. FMT-CUMS+SCFAs	0.0010	0.0051	0.0007	<0.0001
FMT-CDK vs. FMT-CDK+GLPG0974	0.0012	>0.9999	0.0866	<0.0001
